# Philadelphia‐negative myeloproliferative neoplasms among Kuwaiti Nationals

**DOI:** 10.1002/cam4.3633

**Published:** 2020-12-06

**Authors:** Salem Alshemmari, Mazyad Almazyad, Aisha Alwehaib, Reem Ameen

**Affiliations:** ^1^ Department of Medicine Faculty of Medicine Safat Kuwait; ^2^ Sixth Year Medical Students Faculty of Medicine Kuwait University Safat Kuwait; ^3^ Department of Medical lab Sciences Faculty of Allied Health Safat Kuwait

**Keywords:** driver mutations, epidemiology, Myeloproliferative neoplasms

## Abstract

The epidemiology, genetics, and thrombosis risk of MPNs among Arabs are largely unknown. This may be attributed to scarce epidemiological data, particularly from our region. Our study included 381 Kuwaiti nationals with Ph‐negative MPNs and a confirmed driver mutation involving JAK2 (exon 12 14), CALR, or MPL. This first regional study examines the demographics, clinical parameters, and thrombosis‐related attributes of the participants. This study reported a median age of 58 years, with females and males representing 54.9% and 45.1%, respectively. ET was the most frequent subtype of Ph‐negative MPNs in our population, accounting for 52.0% of the cases, followed by PV, found in 34.6% of the participants, and PMF, found in 8.4% of participants. The crude annual cumulative incidence of Ph‐negative MPNs in Kuwait ranged from 0.674 to 3.177 per 100,000 population across the study period. The most common driver mutation was JAK2V617F, with a frequency of 89.5%. At diagnosis, 19.2% of the patients presented with unexplained thrombosis, and almost half were of arterial origins. Males were more likely to present with arterial thrombosis than females (61.5% vs. 35.3%), whereas venous thrombotic events were more common in females than in males (47.1% vs. 17.9%; *p*‐value = 0.025). Ph‐negative MPNs in Kuwait are rare; however, thrombosis is a frequent complication, being documented in up to 19.2% of cases at presentation, more commonly at arterial sites. These findings call for thorough evaluation of patients with unexplained derangements in their hematological parameters during follow‐ups.

## Graphical abstract

This is a unique study that examined the epidemiology of Myeloproliferative Neoplasms among Arabs.

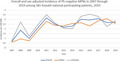

## 1 INTRODUCTION

Myeloproliferative neoplasms (MPNs) are a group of diseases characterized by clonal hematopoiesis with overproduction of mature cells from erythroid, megakaryocytic, and myeloid lineages.^1^ These clonal stem cell disorders were first conceptualized in 1951 by William Dameshek, and they historically included the Philadelphia (Ph) chromosome‐positive chronic myeloid leukemia (CML), and the Ph‐negative polycythemia vera (PV), essential thrombocythemia (ET), and primary myelofibrosis (PMF).^2^ Disease‐specific genetic abnormalities have not been detected that distinguish PV, ET, and PMF until recently.^3^ In 2005, a point mutation in Janus kinase 2 (JAK2V617F) was described as a driver mutation in MPNs by multiple scientific groups.^4^, ^5^ This discovery was followed by a series of additional descriptions of mutations that directly or indirectly activate the JAK‐STAT pathway: JAK2 exon 12, myeloproliferative leukemia virus oncogene (thrombopoietin receptor; MPL)^6^ and calreticulin (CALR) mutations.^7^, ^8^, ^9^ Accordingly, the 2008 WHO classification has incorporated these discoveries into the diagnostic criteria of MPNs, which were further refined in 2016.^10^ As the only referral cancer center in Kuwait, we are receiving all the samples with suspected MPN from different hospitals in the country. In 2006, after the discovery of JAK2V617F and its diagnostic value in MPNs, we established an MPN molecular referral laboratory. The aim of this study is to report the demographic features, clinical parameters, incidence, and thrombosis‐related attributes of Kuwaiti Ph‐negative MPN cases with documented driver mutations reported from January 2007 to December 2019.

## 2 METHODOLOGY

We received 5290 samples from various hospitals in Kuwait with suspected MPN and elevated blood counts.

Extraction of Genomic DNA: Blood samples were collected from each subject in vacutainer tubes containing 1.8 mg/ml of K2 EDTA. The extraction of total genomic DNA was carried out following the protocol of the QIAamp^®^ Blood Mini Kit (Qiagen).

### 2.1 ARMS‐PCR

An ARMS‐PCR assay was used in this study for the initial detection of the JAK2V617F mutation. The ARMS‐PCR assay uses two primer pairs to specifically amplify the normal (229 bp) and mutant (279 bp) sequences plus a positive control (463 bp) in a single reaction. One hundred nanograms of DNA template and HotStart Taq Polymerase Master Mix (Qiagen) were used for amplification. Steps for thermal cycling conditions were denaturation at 94°C for 1 minute, annealing at 58°C for 40 seconds, and extension at 72°C for 45 seconds. Products were visualized on a 1.5% of agarose gel after staining with ethidium bromide.

### 2.2 MPL analysis

Allele‐specific real‐time PCR was performed in this study for the detection of mutant MPL515 L/K cells. A standard MPLW515L/K MutaScreenTM Kit (Ipsogen, 676413) containing the MPL 515L/K mutant positive control, negative control, and a reference sample was used for the discrimination of positive and negative cells. Genomic DNA samples were diluted in TE buffer (Ambion) to 5.0 ng/µl. For the amplification of the mutant fragment, TaqMan Universal Master Mix (Applied Biosystems) was added to the primer/probe mixture (MPL 515L/K wild‐type primer/VIC probe, MPL515L/K mutant primer/FAM probe).

### 2.3 CALR analysis

Patients were screened for CALR mutations by high‐resolution sizing of fluorescence‐labeled PCR products by capillary electrophoresis (fragment analysis). For the detection of CALR mutations, 20 ng of genomic DNA was amplified with 10 pmol each of forward and reverse primers. For fragment analysis, PCR was carried out with 6‐FAM‐labeled forward primers in AmpliTaq Gold 360 Master Mix (Applied Biosystems). Products were separated by electrophoresis on a 2% of agarose gel and analyzed by capillary electrophoresis on an ABI 3130 genetic analyzer followed by fragment analysis by GeneMapper Software 4.1 (Applied Biosystems).

### 2.4 Statistical analysis

Data entry and analysis were accomplished by using Statistical Package for the Social Sciences (SPSS). Statistical analysis involved computation of measures of central tendency and dispersion (mean, median, standard deviation, and interquartile range). Pearson Chi‐Square test and Fisher's Exact test were used to assess the statistical significance of associations between categorical variables. A *p*‐value < 0.05 was considered significant.

## 3 RESULTS

### 3.1 Demographic features of participating patients

The study, which encompassed a period from 2007 to 2019, involved a total of 381 Kuwaiti enrollees who tested positive for Ph‐negative MPN‐related driver mutations. Table 1 demonstrates that among the participants, 137 (36.5%) patients were 40–59 years of age and 174 (46.4%) patients were 60 years or older. The reported mean (±SD) and median (±IQR) ages of the 381 enrollees were 56.46 (±16.98) and 58.00 (±25.00), respectively. In terms of sex, Table 1 reports that 209 (54.9%) participants were females, and the rest of the patients were males (172; 45.1%).

**TABLE 1:** Demographic features and clinical attributes of 381 Kuwaiti national participating patients, 2019.

Characteristic	Frequency	
	n	(%)
Age at diagnosis^a^		
0–19	9	(2.4)
20–39	55	(14.7)
40–59	137	(36.5)
≥60	174	(46.4)
*Mean Age (±SD)*	*56.46*	*(±16.98)*
*Median Age (±IQR)*	*58.00*	*(±25.00)*
Sex		
Male	172	(45.1)
Female	209	(54.9)
Diagnosis		
PV	132	(34.6)
ET	198	(52.0)
PMF	32	(8.4)
MPN‐Undetermined	6	(1.6)
Post‐MPN AML	1	(0.3)
Post‐ET/PV MF	12	(3.1)
Driver mutations		
JAK2 V617F Positive	341	(89.5)
CALR Positive	35	(9.2)
MPL Positive	5	(1.3)
JAK2 Exon 12 Positive	0	(0.0)
Thrombotic events		
Yes	73	(19.2)
No	308	(80.8)
Site of thrombosis		
Arterial	36	(49.3)
Venous	23	(31.5)
Both	1	(1.4)
Unregistered	13	(17.8)

^a^ The total does not add up to 381 owing to missing data.

### 3.2 Incidence of PH‐negative MPNS among participating patients

**FIGURE 1:**
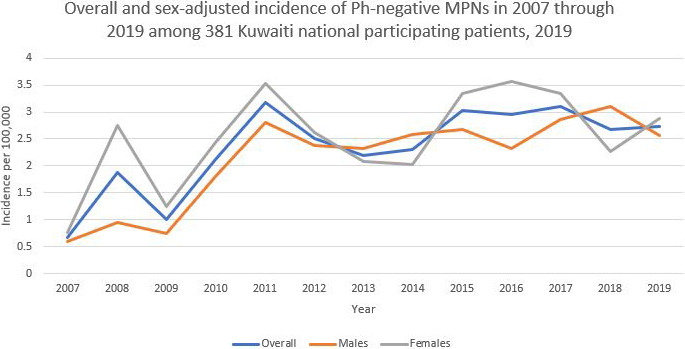
Overall and sex‐adjusted incidence of Ph‐negative MPNs from 2007 to 2019 among 381 Kuwaiti national participating patients, 2019

**FIGURE 2:**
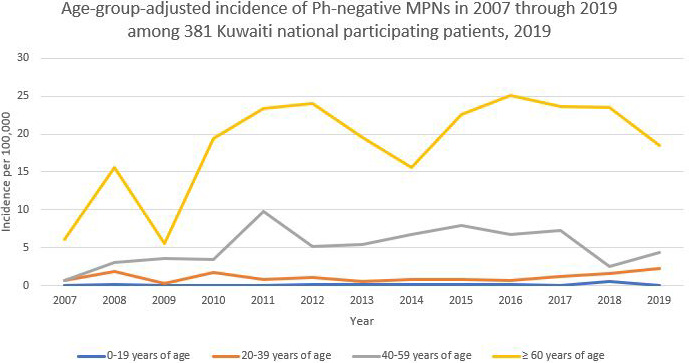
Age‐group‐adjusted incidence of Ph‐negative MPNs from 2007 to 2019 among 381 Kuwaiti national participating patients, 2019

A general increasing trend in the cumulative incidence (per 100,000 population) of Ph‐negative MPNs was observed among the 381 Kuwaiti participants from 2007 to 2019 (Figure 1). It was also shown that the highest overall incidence among the enrollees was 3.177, documented in 2011. After adjustment for sex, females generally showed a higher incidence of Ph‐negative MPNs across the study period than males. The highest incidence documented among females was 3.564 in 2016 (Figure 1). Moreover, adjustment for age groups demonstrated that patients who were 60 years of age or older generally exhibited the highest incidence over the study period (Figure 2). Furthermore, an increased incidence of Ph‐negative MPNs is reported with an increase in age across the study period, as shown in Figure 2.

### 3.3 Clinical attributes and parameters of PH‐negative MPNS among participating patients

Among the 381 Kuwaiti participants, 198 (52.0%) were diagnosed with ET, followed by PV, found in 132 (34.6%) participants, and PMF, found in 32 (8.4%) participants (Table 1). The cases of six patients (1.6%) were classified as MPN‐undetermined, and 12 (3.1%) participants were diagnosed with post‐ET/PV myelofibrosis (MF), in addition to a single patient (0.3%) carrying the diagnosis of post‐MPN acute myeloid leukemia (AML). In terms of driver mutations, most individuals (341; 89.5%) tested positive for the JAK2V617F mutation, while those who tested positive for CALR mutation and for MPL mutation represented 35 (9.2%) and 5 (1.3%) participants, respectively. No positive result for the JAK2 exon 12 driver mutation was reported across the study period (Table 1). The analysis of the clinical parameters in this study included the mean hemoglobin (Hb) level (g/L), white blood cell (WBC) count (×10^9^/L), and platelet (Plt) count (×10^9^/L), according to MPN phenotypes, as shown in Table 2.

**TABLE 2:** Clinical parameters of 381 Kuwaiti national participating patients at presentation, according to diagnosis, 2019

Clinical parameter	PV		ET		PMF	
Hb (g/L)						
Mean (±SD)	158.8	(±24.3)	130.7	(±18.4)	107.0	(±22.3)
**WBC (×10^9^/L)**						
Mean (±SD)	12.1	(±8.7)	10.0	(±5.2)	15.6	(±12.6)
**Plt (×10^9^/L)**						
Mean (±SD)	470.4	(±218.1)	717.8	(±274.3)	439.0	(±325.6)

### 3.4 Descriptive characteristics of thrombosis cases among participating patients

The frequency of thrombosis among the 381 Kuwaiti Ph‐negative MPN patients was 73 (19.2%) cases, as demonstrated in Table 1. Out of these thrombotic events, 36 (49.3%) patients had an arterial‐site thrombosis, 23 (31.5%) cases were of venous origin, and a single (1.4%) participant had a thrombotic event arising from both arterial and venous territories (Table 1). Table 3 illustrates that PV and ET were the most common types of Ph‐negative MPNs presenting with thrombosis, as well as that most of these thrombotic events occurred in those with advanced age. The relationship between sex and both the frequency and the site of thrombotic events is examined in Table 4, revealing that cases of thrombosis occurred in males more than in females, although the difference was statistically insignificant. Moreover, males were shown to experience more cases of arterial thrombosis (24; 61.5%) than females (12; 35.3%). In addition, females had a higher frequency of thrombotic events of venous origin (16; 47.1%) than males (7; 17.9%) (*p*‐value = 0.025).

**TABLE 3:** Thrombosis and associated attributes among 381 Kuwaiti national participating patients, according to diagnosis and age, 2019

Characteristic	Frequency of Thrombosis	
	n	(%)
Diagnosis		
PV	38	(52.0)
ET	27	(37.0)
PMF	7	(9.6)
MPN‐Undetermined	0	(0.0)
Post‐MPN AML	0	(0.0)
Post‐ET/PV MF	1	(1.4)
Age		
0–19	0	(0.0)
20–39	9	(12.3)
40–59	28	(38.4)
≥60	36	(49.3)

**TABLE 4:** Association between sex and the occurrence of thrombotic events and the site of thrombosis among 381 Kuwaiti national participating patients, 2019

Clinical Outcome	Male		Female		*p*‐value
	n	(%)	n	(%)	
Thrombotic events					0.114^a^
Yes	39	(22.7)	34	(16.3)	
No	133	(77.3)	175	(83.7)	
Site of thrombosis^b^					0.025^c^
Arterial	24	(61.5)	12	(35.3)	
Venous	7	(17.9)	16	(47.1)	
Both	1	(2.6)	0	(0.0)	

^a^ Pearson chi‐squared test.

^b^ “Unregistered” group was not included in the table.

^c^ Fisher's exact test was used since two cells (25.0%) had an expected count of less than 5.

## 4 DISCUSSION

This study is the largest population‐based study in the Middle East that examined the demographic features, clinical parameters, and driver mutation profiles of Ph‐negative MPNs at presentation. The data used in our study were obtained from the only molecular laboratory that conducts myeloid diagnostics in Kuwait.

Our results demonstrated that the crude annual incidence of all Ph‐negative MPNs among Kuwaitis over the past 13 years ranged from 0.674 to 3.177 per 100,000 population. These findings are similar to the meta‐analysis results published by *Titmarsh* et al., who reported the crude annual incidence rate of Ph‐negative MPNs to be between 1.15 and 4.99 per 100,000.^11^ There are wide variations in the incidence of PV, ET, and PMF across the globe, which can be attributed to disease classification changes, differing etiological exposures, and the limitations of cancer registration around the world.^11^ Additionally, the underreporting of specific entities among MPNs, such as ET, has been documented in the literature.^12^ However, this issue is not relevant in our study, as we are the only referral center for MPN diagnostics in the country, thereby ensuring our data carries a significant level of representativeness.

Moreover, the incidence of Ph‐negative MPNs has increased steadily over the past 13 years, which is in line with worldwide published studies. This can possibly be explained by the introduction of molecular diagnostics and the implementation of the 2008 WHO classification of MPNs and its revised 2016 version.^13^, ^14^ Furthermore, we believe that the increasing awareness of general practitioners of the new diagnostic criteria and MPN driver mutations has also contributed to this observation. Additionally, we speculate that the incidence at the beginning of the study might have been affected by the case pool. Some cases that were left undiagnosed prior to the introduction of the 2008 WHO MPN diagnostic criteria may have been diagnosed later as cases of MPN, overestimating the true incidence to some degree. This effect could be minimized by evaluating the incidence over a long enough period, which we believe was achieved by carrying out our study over 13 years. It is also important to highlight the fact that the increase in incidence might be partially explained by the aging of the Kuwaiti population.

In terms of age, our study participants had a median age at diagnosis of 58, which is younger than the reported median age in the literature. Several studies reported the median age at diagnosis to range from 69 to 76 years.^15^, ^16^, ^17^, ^18^ This finding might be attributed to the fact that the Kuwaiti population is generally younger than the world's general population. Previous studies have demonstrated that the reported incidence of Ph‐negative MPNs is increasing with age, which ties well with our results. It has been suggested by *Srour* et al. that this increase is due to accumulating DNA damage, immunosenescence, and autoimmunity that predispose older people to acquire mutations leading to the development of MPNs.^19^

Among the 381 Kuwaiti Ph‐negative MPN patients, 19.2% presented with thrombosis, with an increased prevalence among PV patients, followed by ET patients. Overall, these results are in accordance with the findings reported by *Szuber* et al and *Rungjirajittranon* et al.^20^, ^21^ Among those who presented with thrombosis, nearly half of them were arterial in origin. This is close to what *Arachchillage and Laffan* published, which reported the prevalence of arterial thrombosis to be above 60%.^22^ In addition, the relationship between sex and the site of thrombosis showed statistical significance, in which arterial events were more common in males and venous events were more common in females. Furthermore, the majority of these thrombotic events occurred in older patients, implicating the possibility of a role played by other risk factors common in this age group, such as cardiovascular diseases. Thrombosis is considered a major cause of morbidity and mortality among patients with MPNs, and studies have shown that intervention with lifelong oral anticoagulants reduces this risk by 48–69%.^23^

## 5 LIMITATIONS

This population‐based study also has limitations. Due to the registry‐based nature of our data, we lack detailed documentation on treatment and interventions. Furthermore, we did not include any individuals without driver mutations, and there was a lack of proper registration in the general hospitals from which these samples were obtained. However, such cases are rare and may not affect the general epidemiological picture of Ph‐negative MPNs in Kuwait. Moreover, this study does not include additional epidemiological measures, such as survival rates.

## 6 CONCLUSION

In summary, Philadelphia chromosome‐negative myeloproliferative neoplasms are considered rare myeloid malignancies in Kuwait, with their incidence showing an upward trajectory but still consistent with worldwide results. Up to 19.2% of such MPN cases are diagnosed during an episode of unexplained thrombosis. The evidence from this study also showed that males diagnosed with Ph‐negative MPNs are more likely to develop thrombosis of arterial origin, whereas females carrying a diagnosis of MPNs tend to develop venous‐site thrombosis. Such results would provide the first step in tackling thrombotic events associated with MPNs to prevent their occurrence. In addition, these observations should warrant a more thorough evaluation of MPNs to provide a better understanding of its epidemiology, as well as raising further awareness among general practitioners to promptly refer suspected cases of Ph‐negative MPNs.

## CONFLICTS OF INTEREST

The authors have no conflict of interest to declare. Data could be available upon request to the corresponding author.

## AUTHORS’ CONTRIBUTIONS

Conception and design: Salem H Alshemmari, Reem Ameen; Provision of study materials or patients: All authors; Data management and Statistical Analysis: Mazyad Almazyad, Aisha Alwehaib; Interpretation: Salem Alshemmari, Reem Ameen, Mazyad Almazyad; Manuscript writing: Salem Alshemmari, Mazyad Almazyad, Reem Ameen; Final approval of the manuscript: All authors; Accountable for all aspects of the work: All authors.

## Data Availability

Data subject to third party restrictions
